# Shared Book-Reading in Early Childhood Education: Teachers’ Mediation in Children’s Communicative Development

**DOI:** 10.3389/fpsyg.2020.02030

**Published:** 2020-09-08

**Authors:** Karina Cárdenas, Ana Moreno-Núñez, Edgardo Miranda-Zapata

**Affiliations:** ^1^Departamento de Pedagogía en Educación Parvularia, Pontificia Universidad Católica de Chile, Campus Villarrica, Villarrica, Chile; ^2^Departamento de Psicología Evolutiva y de la Educación, Universidad Autónoma de Madrid, Madrid, Spain; ^3^Laboratorio de Investigación en Ciencias Sociales Aplicadas, Universidad de La Frontera, Temuco, Chile

**Keywords:** shared book-reading, early childhood education, infant development, teaching practices, triadic interaction, communicative development

## Abstract

Fostering communicative skills in young children is essential for their holistic development. Book-reading activities have been shown to be a valuable tool for supporting communicative exchanges between children and adults, but there is limited research on actual educational practices with children under 3 years old. This experimental study explores teaching practices in Chilean early childhood education with children from 4 to 17 months of age. We focused on children’s performance of diverse communicative signs, as well as on the effect of the teacher’s mediation (signs and strategies) in a triadic shared-reading interaction (teacher-child-book). The study is part of a larger cross-sectional project. We conducted an experimental study following a pre-test–post-test design with 11 children, who were randomly assigned to either the control or the experimental group. In addition, we conducted a 6-week intervention on shared book reading between the pre- and post-test stages. We observed that children used a wide range of communicative signs when engaging in shared interactions with their teacher and different books. In the experimental group, children performed more communicative signs after participating in the intervention than at the beginning of the study. The reading experience that they gained through the intervention could also explain the larger proportion of uses of the books, as compared to their control counterparts. Additionally, children performed different combinations of vocalizations, words, or repetitions within a single use. The conventional use of a book is not evident for an infant, and as such it requires the systematic and semiotically mediated action of an adult to be consolidated. We conclude that offering preschool teachers a diverse selection of books enables them to better adjust to the particularities of each child. In this scenario educators are able to promote efficient spaces for children’s participation, increasing the complexity and variety of their communicative repertoire.

## Introduction

Language is a culturally located artifact. That means that it is dynamically integrated into a complex system of meanings that includes other symbolic and material artifacts (Sinha, 2015). This makes language an important cultural product (Tomasello, 1999), and as such it is cognitively and semiotically complex. As occurs with other artifacts, language serves diverse canonical functions and is implicated in a wide range of cultural practices, including representation and narration. Children get used to these canonical functions through their participation in social interchanges with adults and peers (Estrada and Cárdenas, 2017; Moreno-Núñez et al., 2017). Likewise, the gradual mediation of others helps children to learn how to use several objects and instruments, since they imply rules of use that are not evident, especially for young infants. The role of adults as mediators between the child and the world has been widely emphasized from the Pragmatics of the Object perspective (Rodríguez and Moro, 1999), moving the Vygotskyan discourse to younger ages. From this perspective, adults’ mediation allows children to get involved in increasingly complex triadic interactions (adult-child-object). This facilitates children’s building their relationship with the world in interactive niches and in getting used to diverse semiotic systems (such as gestures, or the conventions around the use of objects) during their first two years of life (Rodríguez et al., 2018).

Children need opportunities to participate in educational-communicative interactions with others to learn how to use objects according to their social function. This is a gradual cultural appropriation that starts from birth, when infants are typically introduced into communicative niches mediated by the intentional action of adults (Rodríguez et al., 2017, 2018). Thanks to this semiotic mediation, children gradually abandon their initial undifferentiated, non-canonical or unconventional uses (e.g., sucking, throwing, or banging the object) on their way to learning the cultural uses that are relevant for their community (Rodríguez and Moro, 1999), and so allow them to share common grounds with others (Dimitrova, 2020; Dimitrova and Moro, 2013). Knowing the rules of use that are shared by the community is a process that requires gradual approaches to the object, in which adults play a crucial role.

Given its importance, fostering children’s communicative abilities is one of the core learning goals to be acquired throughout early childhood education (ECE) according to the Chilean Guiding Principles of the Curriculum (Ministerio de Educación, 2018). In this sense, some educational practices, such as shared book-reading, become privileged opportunities to foster the social and cultural construction of cognitive and communicative development (Munita, 2010; Rossmanith et al., 2014). Thus, promoting the availability and accessibility of book-related activities and materials could be of great relevance for ensuring better classroom-based practices in ECE.

This study explores classroom-based, shared book-reading and teachers’ mediation with Chilean babies up to 18 months old by focusing on their developmental changes. We focus on how children typically interact with their teacher during shared book reading interactions and analyze their production of communicative signs—i.e., uses of objects (books), gestures and vocalizations—before and after participating in a 6-week intervention aimed at promoting reading practices in ECE. The study adds to the very limited classroom-based international literature on book reading activities with children under 3 years old (Contin and Rodríguez, in press).

### The Importance of Adult Mediation and Communicative Exchanges

Psychological research on prelinguistic communication has shown that infants become able to establish shared referents in interaction with others during the second half of their first year (Murillo and Belinchón, 2013). They gradually learn to understand and perform gestures from their everyday experience with others. At the beginning, children communicate by using those signs that are linked to materiality, or through ostensive gestures in which the object occupies the hand (Rodríguez et al., 2015). Progressively, they start to perform signs that refer to other aspects beyond the boundaries of the “here and now,” forming a solid basis for the later use of words (Solís et al., 2016). The repertoire of prelinguistic communicative resources that children acquire during this stage coalesce into their first verbal utterances as they develop more complex linguistic structures.

In this scenario, reading has been shown to be an impactful means for increasing the amount and content of children’s conversations, thereby enhancing early language development. Reading is a social practice that greatly contributes to meaning comprehension and production in human communication (Reyes, 2007). In early childhood development, interactions based on non-written books (i.e., picture books) are more likely to favor verbal behaviors from parents and infants, than those with books in which there is a fixed text to follow (Sénéchal et al., 1995). Moreover, reading increases the variety of vocabulary and extends the extratextual sentences that children become able to produce (Hammett et al., 2003). Thus, shared book reading may foster important communicative abilities in children, such as phonological awareness, speech fluency, and connections with previous knowledge, that together establish the basis for language comprehension and vocabulary acquisition (Suárez et al., 2018).

But infants’ entry into such complex conventions and practices cannot be fully understood without analyzing the mediating role of adults. Adults’ mediation facilitates children’s first encounters with cultural meanings (Munita, 2010), in addition to contributing to the conveying of emotions (Sainz, 2005) that turn the story told into a meaningful book reading experience. Recent studies have shown that longitudinal follow-ups are a fruitful approach for the investigation of the role of adults in shared book reading activities. They allow for a thorough analysis of the communicative signs that both children and adults use during triadic interactions with books, either in one-to-one or whole-group settings. A good example of this is the longitudinal study conducted by Rossmanith et al. (2014), in which researchers observed children from 3 to 12 months of age in interaction with their parents and children’s books. Their results showed that adults stimulated their children while reading the books by using diverse communicative signs (including gestures and affective interactions). They promoted triadic spaces for purposeful shared actions with the babies, using specific and increasingly complex language as the children grew up. Children at 6 months of age were already able to explore the books by themselves and to produce their own communicative signs. For example, they often directed their gaze toward their mother or used affective cues (e.g., smiles) and vocalizations to get her attention during the shared interaction.

Findings in home settings and parent-children interactions have important implications that could be transferred to the ECE classroom, since children’s communicative competence is one of the main learning objectives of this educational stage. In a time when infants are schooled at an increasingly younger age, acquiring basic cognitive and communicative milestones, it is essential to consider adults’ mediation because of its impact on children’s present and future development.

### Book Reading Activities as an Educational Tool

It is known that shared book reading brings great benefits for the cognitive and communicative development of the child. It favors the affective bonds between the participants (Swartz, 2010), as well as promoting the learning of new vocabulary (Hargrave and Sénéchal, 2000) and varied communicative styles such as the poetic and the narrative. In addition, reading fosters the infants’ knowledge of their particular and social environments (Colomer, 2005; Colomer and Durán, 2008), which in turn contributes to children’s holistic development and the construction of the self (Munita, 2010).

Several studies have investigated how sharing picture books with young infants can benefit language development. Since parent-child book-based interactions seem to be a very effective tool for fostering cognitive and communicative development (Goldstein et al., 2016; Rossmanith et al., 2014), specific book sharing interventions have been aimed at training parents on how to promote this type of activity when interacting with their children (Hardma and Jones, 1999; Hargrave and Sénéchal, 2000). In a recent meta-analysis, Dowdall et al. (2019) showed that there is a small but positive effect from these interventions on children’s development of both expressive and receptive language. Substantial evidence from these interventions also suggests that the sharing of books also contributes to children’s emergent literacy and reading achievements (Bus et al., 1995). However, despite this being a continuing topic of interest in developmental psychology, the evidence available on book sharing interactions in ECE settings is limited (Valdez-Menchaca and Whitehurst, 1992).

Children’s first contacts with the cultural practice of reading are typically produced through oral forms of language (Colomer, 2005). Adults can scaffold and support the communicative development of children during their first year of life (Hoff, 2010; Rogoff, 2003) as infants can be easily engaged with simple stories and texts. While there is evidence that the type of book that is read is associated with differences in adults’ reading styles (Pellegrini et al., 1990; Reese and Cox, 1999), there is little literature on how preschool educators use reading-based educational practices with young children. In an observational study examining reading patterns of early childhood educators of infants from 4 to 27 months, Honig and Shin (2001) noted that they rarely read to very young babies. Teachers’ reading practices were rather brief and based mainly on one-to-one interactions, even with toddlers over one year of age. The authors conclude that teachers’ professional development courses should include developmental contents that spur them to foster book-sharing interactions with young babies. It is crucial to observe children’s responses while they engage in shared book reading activities (Lamme and Packer, 1986) in order to carefully adapt the interaction to their competencies and general development (Copple and Bredekamp, 2009). For instance, when there are signs of disengagement during a shared action, it is recommended to quickly switch to a different topic or to conclude the interaction, rather than persisting with the activity.

These ideas point to the need for adults to carefully choose the reading materials for each infant, planning the learning goal of the activity before it starts. As Tare et al. (2010) suggest, books with photographs or realistic illustrations facilitate learning in children under 3 years old, unlike manipulative books (i.e., pop-ups) which are more appropriate for leisure at this age. During the first and second years of life, experts recommend books with clear and eye-catching images, and those with a story that refers to children’s familiar context and daily routines (Lluch, 2010). Also, books based on rhymes where repetition is a key factor are particularly appropriate, as well as those about animals and objects with which the child can interact (Rosenquest, 2002). While adults initially select the books for children, infants should also have individual experiences with books, as it allows them to make their own choices and to build up their personal aesthetic taste (Munita, 2010). Before the end of the first year, children start to show their personal preferences, usually related to more elaborated narratives, both text and image-based (Bonnafé, 2008). But, as children’s interest on books depends on their reading experience (Reyes, 2007), they should also be provided with enough time and opportunities for using the books while in the classroom (Estrada and Cárdenas, 2017).

In the past decades, scholars and policy-makers have emphasized the low value in which reading habits are held in Chile and an increasing weakening of family interactions. According to Ministerio del Trabajo y Previsión Social (2012) social inequalities in Chile originate in early childhood and in the sociocultural context in which children develop. The proportion of book readers is strongly associated with the household socioeconomic status (CNCA, 2015) and the family environment is one of the main settings where the child meets with books and reading habits. The current circumstances require, then, consistent policies aimed at reducing these inequalities. In order to guarantee children’s access to books and reading practices in ECE settings (Sainz, 2005), both governmental and private bodies in Chile have developed specific policies intended to guarantee reading as an essential factor in the education of creative, reflective, and participative citizens. For instance, the program *“Nacidos para Leer”* [born to read] was devised under the National Plan for the Promotion of Reading and the National Policy for Reading and Books (2015–2020), which acknowledges the significance of the accessibility of books as a universal right for children.

These programs are aligned with the learning objectives that the Chilean Guiding Principles of the Curriculum (Ministerio de Educación, 2018) established for the communicative development of early preschool children (0–3 years old). Teachers are encouraged to create spaces for initiating infants into communicative interchanges through gestures, body movements, and other prelinguistic signs that could help the infants to progressively increase the complexity of their own communicative resources. While previous studies have raised the importance of enacting constructive forms of communicative interactions during preschool (Bautista et al., 2018), there are few examples in the international literature of literacy initiatives for increasing the frequency of book sharing. Additionally, early childhood educators need information and support for engaging in book reading activities with very young babies, mediating their development through varied strategies and means that allow children to acquire various kinds of knowledge, such as the meaning of the pictures and book-reading conventions (Hardma and Jones, 1999). Educational and mediated practices in preschool need to draw on evidence-based research, for which Chilean investigators have been called to produce new and contextualized knowledge (Munita, 2010).

In this study, we look at teachers’ mediation, in which they promote triadic interactions with the children and one or more books, using several linguistic and non-linguistic signs (semiotic mediation) and also some specific strategies to mediate the shared reading situation.

Despite the importance of book-related activities in early childhood, there is an important gap in the international literature. First, studies of shared book reading practices in early infancy focus primarily on children’s interactions with their parents (Snow et al., 1976; Ninio, 1980; Holdaway, 1982; Ortiz et al., 2001; Tsuji, 2002; Yont et al., 2003; Karrass and Braungart-Rieker, 2005; Landry et al., 2012) rather than with teachers (Zucker et al., 2009; Tare et al., 2010). Additionally, classroom-based studies are frequently conducted with children over 3 years of age (Martinez and Teale, 1993; Moschovaki et al., 2007; Price et al., 2012; Milburn et al., 2014) while there are few examples of teachers’ mediation practices in early infancy. These trends suggest that the research interest on this topic is not equally distributed (Rutanen, 2007), which is paradoxical as greater brain plasticity occurs during the 0–3 year period (Poch, 2001), setting the basis for subsequent development.

While there is evidence that during the first years of life children are highly competent at participating in triadic interactions with adults and books (Rossmanith et al., 2014; Contín, 2017; Estrada and Cárdenas, 2017), the number of studies that focus on actual educational practices at these ages are few, especially in Latin America. This study responds to the need for evidence-based studies that reveal children’s developmental changes in communication and interaction during shared book reading activities. Our ultimate aim is to contribute to the initial and continuous training of early childhood educators as reading mediators, as well as to guarantee children’s right to access reading by promoting their opportunities in ECE settings.

### Goals

The overall purpose of the present study was to identify the amount and diversity of communicative signs that children use during shared book reading with their teacher. Additionally, the study investigated whether there is an effect of the teacher’s mediation on the children’s production of communicative signs. Analyses were conducted to address the following hypotheses: (1) the production of communicative signs would not differ between children in the experimental and control groups during the pretest stage; (2) the production of communicative signs would be significantly different between children in the experimental and the control groups after the intervention; (3) the production of communicative signs by the control group (CG) would not significantly differ when comparing the pre- and post-test results; (4) the production of communicative signs of the experimental group (EG) would show significant differences in a comparison of the pre- and post-test results.

## Materials and Methods

The study followed a pre-test–post-test design where children were randomly assigned to either the CG or the EG. In addition, we conducted a 6-week intervention on shared book reading between the pre- and post-test stages.

### Materials

#### *Ad hoc* Questionnaire

Parents were asked to complete a brief questionnaire that was devised *ad hoc* for this study. The questionnaire collected sociodemographic information about the parents (e.g., their highest educational level) as well as the children’s reading habits, in order to determine whether or not reading practices were frequent in a particular household.

#### Books

For this study, we used a total of 50 different books, including interactive books, board books, soft books, picture and poetry books, and information books. All books were carefully selected by the researchers according to the following criteria: (1) the appropriateness of the characteristics of the book for gaining young children’s interest, (2) the recommendations made by Lluch (2010) for book selection criteria (i.e., brief and jazzy books with pictures that encourage storytelling and that are related to children’s day-to-day experiences); (3) the book’s suitability for children under 3 years of age, as suggested by Álvarez et al. (2013); and (4) its outstanding literary quality and aesthetic value (Reyes, 2007).

**FIGURE 1:**
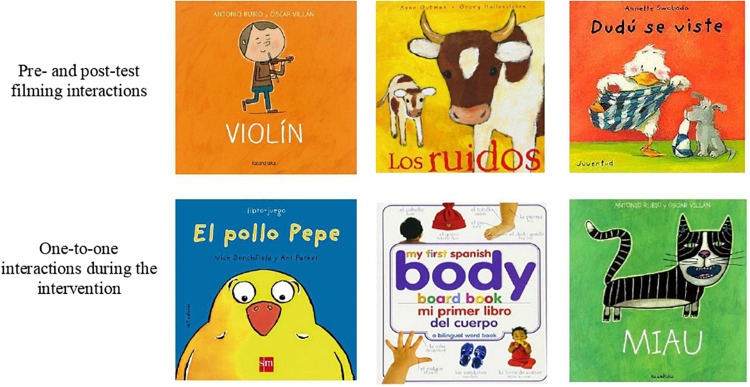
Set of books selected for the study.

Previous research has shown that different types of books could be associated with differences in reading styles and interactions (Pellegrini et al., 1990; Reese and Cox, 1999) as well as in the use of diverse communicative signs during book-based interchanges (Estrada and Cárdenas, 2017). Following these results, we selected a set of three books for the pre- and post-test stages of the study: a narrative book, an informative book, and a poetry book (Figure 1). These three books were not offered during the intervention phase. Instead, we offered the teacher the remaining 47 books for the intervention, so that she could select the ones that she considered the most appropriate for both one-on-one and group reading interactions.

We used two more sets of books for *one-on-one interactions during the intervention*. The first set consisted of three fixed books selected by the research team (Figure 1), while the second set consisted of three books selected by the teacher. The second set could vary every week according to the teacher’s criteria and knowledge about the child with whom she was going to interact (such as personal preferences, developmental stage, or previous experiences). For *group reading sessions during the intervention* the teacher selected three books per session, that is, six books per week. She was free to decide whether or not to repeat the books she read to the children in each session.

#### Infant Learning and Development Test (TADI)

We used the language development subtest of the Infant Learning and Development Test [Test de Aprendizaje y Desarrollo Infantil, TADI] (Edwards and Pardo, 2013) for assessing all participating children twice (first in the pre-test stage and later during the post-test). The language subtest measures both comprehensive and expressive language abilities. It was standardized in Chile, making it appropriate for this particular study as it incorporates the Chilean cultural context. This is reflected, for instance, in the inclusion of relevant items for children regardless of their gender, socioeconomic status, area of residence, or ethnicity.

The TADI is administered individually and is aimed at children aged between 3 months and 6 years old. It has start and stop criteria for several items that are organized in 13 age ranges. This structure allows comparison of the performance of a child with that of their same age counterparts. Raw scores are converted to T-scores for interpretation. A regular administration of this test could help to monitor the children’s development and the evolution of their learning.

The language subtest showed good psychometric properties in terms of concurrent validity [*r* = 0.604, *p* < 0.01 with the Psychomotor Development Scale (EEDP)], construct validity (through exploratory factor analysis that allowed the identification of the Language dimension) and reliability (Cronbach’s Alpha = 0.925).

### Participants and Procedures

We used convenience sampling for selecting one out of the two public early childhood education centers in the city of Villarrica, in southern Chile. The project was first presented to the school leaders and, after they agreed to participate, to the main teacher in charge of a classroom with 0–18—month-old babies. In addition to the teacher, there were also three early childhood education teaching assistants to attend to the 16 infants who were enrolled in this classroom. The children spent 8 h a day at school. Then, we approached all the parents of the 16 children in that classroom resulting in the enrollment of six girls and eight boys in the study. However, during the course of the research, two of the girls moved to a different school, and one boy did not attend data collection sessions during the post-test, so they were all dropped from the study. In total, 11 typically developing children participated in this study (seven males, four females), together with their teacher and three teaching assistants. The project was approved by the Scientific Ethics Committee for Social Sciences, Arts and Humanities of the Pontificia Universidad Católica de Chile (i.e., the Institutional Review Board). We informed the members of the educational team and the parents of the goals and procedures of the study, after which they were invited to voluntarily participate. Informed consents were collected from the teacher, the teaching assistants and the parents before we started data collection.

For group distribution, children were paired according to their age and, whenever possible, because of the sample size, to their TADI language subtest scores and gender (Table 1). The CG was formed by five children (four male, one female), while the EG included six participants (three male, three female). At the beginning of the study children ranged from 4 to 15 months of age (*M* = 10.45; *SD* = 3.98). Their teacher was 29 years old and had four years of teaching experience. However, it was her first year with a class including infants of the age of the youngest babies. Families were unaware of the groups in which their children were placed.

**TABLE 1:** Participating children and ages* at the different times of data collection.

**Control group**			**Experimental group**		
**Participant**	**Pre-test**	**Post-test**	**Participant**	**Pre-test**	**Post-test**
Boy 8	0; 5 (2)	0; 6 (28)	Girl 4	0; 4 (6)	0; 6 (6)
Boy 4	0; 7 (25)	0; 10 (24)	Boy 3	0; 8 (20)	0; 11 (13)
Girl (dropped off)	0; 9 (12)	–	Boy 2	0; 9 (6)	1; 0 (20)
Boy 1	1; 1 (21)	1; 4 (21)	Boy 5	1; 0 (13)	1; 3 (4)
Girl 3	1; 2 (9)	1; 5 (03)	Girl 2	1; 2 (9)	1; 5 (0)
Boy 6	1; 2 (9)	1; 5 (1)	Boy (dropped off)	1; 2 (14)	–
Girl (dropped off)	1; 6 (23)	–	Girl 1	1; 3 (1)	1; 5 (0)

* Age format is expressed in years; months and (days).

Although we have no exact data on family incomes, the ECE center in which we conducted this study is a public school, which means that it preferably attends to children under four years of age and gives priority to those families that require higher government assistance. Table 2 shows the educational level of both parents, as well as some details on the reading habits and quantity of children’s books available at the children’s homes.

**TABLE 2:** Parents responses to sociodemographic details questionnaire (frequency and percentage).

	**Control group**	**Experimental group**
**Educational level of both parents**	***N* = 10**	***N* = 12**
Partial completion of secondary education	3(30.0%)	1(8.3%)
Secondary education	1(10.0%)	5(41.7%)
Partial completion of Technical degree	0(0.0%)	1(8.3%)
Technical degree	2(20.0%)	1(8.3%)
Partial completion of Professional degree	3(30.0%)	1(8.3%)
Professional degree	1(10.0%)	3(25.0%)

**Number of children’s books per household**	***N* = 5**	***N* = 6**

None (they looked for them on the internet)	1(20.0%)	0(0.0%)
1 to 5 books	1(20.0%)	6(100.0%)
6 to 10 books	0(0.0%)	0(0.0%)
11 to 20 books	2(40.0%)	0(0.0%)
More than 20 books	1(20.0%)	0(0.0%)

**Frequency of shared reading instances at home**	***N* = 5**	***N* = 6**

Everyday	2(40.0%)	0(0.0%)
Three times a week	1(20.0%)	2(33.3%)
During the weekends	0(0.0%)	2(33.3%)
Once a month	2(40.0%)	2(33.3%)
Never	0(0.0%)	0(0.0%)

The study consisted of three phases conducted in the following order: pre-test stage, intervention stage, and post-test stage.

#### Pre-test Stage

The pre-test stage had a duration of two weeks. During this period, we asked the parents to complete the sociodemographic details’ questionnaire, while a researcher went to the ECE center to conduct the assessment sessions with the TADI language subtest. At the same time as the TADI was administered, the children were videotaped once in a one-on-one interaction with their teacher and the pre-test set of books. All filming was conducted in the same room in which other educational activities typically take place. The teacher sat on a mat and usually placed the child on her lap. We did not establish a fixed duration for each video *a priori*, to ensure flexibility for adjusting to each child’s interests and attention/participation levels. The teacher was instructed to conduct a shared reading interaction as she would typically do. Meanwhile, the rest of the group participated in their daily activities in another room with the teaching assistants.

Once the pre-test filming was completed, prior to the start of the six-week intervention, the teacher received 8 h of training (over one week) from one of the research team. The training sessions took place at the ECE center after school hours. This training was not solely focused on reading practices, but also on highlighting the importance of triadic interactions in instances of book reading. Reading is simultaneously a social and an educational practice, and as such it helps to promote both cognitive and communicative development. The training addressed the three elements involved in a triadic interaction: children-books-teacher. First, we addressed the most relevant milestones of communicative development in the first two years of life. We focused on the emergence of language as the sign par excellence of human communication, but also on other non-linguistic signs that children typically use to communicate before language (e.g., types of gestures and their functions). We also drew on different criteria for book selection, both literary and non-literary, for children under 3 years of age. Finally, we discussed the role of the teacher as a mediator of reading and communicative practices. We presented some specific strategies for mediating the reading interactions and developing book-based activities, such as connecting the information in the book with the child’s daily experience, demonstrating the conventional use of the book (opening it, turning the pages), telling the story for the child based not only on the text but also on the images, using gestures to accompany the uses of the book, and singing songs that refer to the characters in the book.

#### Intervention Stage

After the pre-test stage, we implemented a six-week intervention according to the criteria of the studies conducted by Valdez-Menchaca and Whitehurst (1992) and Reese and Cox (1999). Children in the EG participated in six one-on-one reading sessions and in 12 group-reading sessions (twice a week) with their teacher. One of the teaching assistants also joined the group reading sessions since they play a crucial support role, especially by providing pedagogical support to the teacher during daily activities. Over the six-week period we ensured that every child participated in at least one individual session (one on one) and two group sessions.

During the intervention, we asked the teacher to conduct the shared book reading interactions according to the training given during the previous stage (i.e., it is unnecessary to read the full book, the importance of linking the contents to children’s previous experiences, she should not insist in reading when the child disengages, the importance of favoring gesture production). We allowed an extra week for filming those children that were absent for one or more intervention sessions due to health-related issues. Table 3 shows the number of intervention sessions that each child in the EG attended.

**TABLE 3:** Number of shared book reading sessions received by each participant in the experimental group.

**Participant**	**One-on-one sessions**	**Group sessions**
Boy 2	5	6
Boy 3	6	7
Boy 5	5	10
Girl 1	6	10
Girl 2	6	12
Girl 4	6	12

For group reading sessions the children sat on the floor in a semicircle. The teacher chose three books for each of these sessions, in which she read and showed the images to the children, inviting them to participate in the interaction. The teaching assistants held the youngest children on their laps, while supporting the teacher’s reading by making comments and references to the text and/or responding to the teacher’s interventions. There was a total of 12 group reading sessions conducted within this study, with a duration that ranged from 7 to 17 min (*M* = 10.25 min) (Table 4).

**TABLE 4:** Duration (in minutes) of shared book reading sessions per participant.

	**Control Group**					**Experimental Group**					
	**Boy 8**	**Boy 4**	**Boy 1**	**Girl 3**	**Boy 6**	**Girl 4**	**Boy 3**	**Boy 2**	**Boy 5**	**Girl 2**	**Girl 1**
Pre-test	6	7	16	12	4	3	11	9	11	4	8
Post-test	4	7	6	9	5	5	7	11	12	11	6

During the six-week intervention, the reading sessions were conducted in the mornings, during one of the time slots usually allocated for outdoor motor development activities. While the intervention was being implemented, all children in the CG continued with the activities that were scheduled for that day under the lead of the teaching assistants. If there were any individual or group reading interventions scheduled, children in the EG went with the teacher to their classroom for the session to be conducted. Children in the CG also received the intervention from their teacher, but at the end of data collection.

#### Post-test Stage

The post-test stage started immediately after completing the intervention, and lasted for two more weeks as in the pre-test stage. Children were reassessed through the TADI language subtest and videotaped again while interacting with their teacher and the same set of three books that were used during the pre-test stage.

### Analysis of Data

#### Coding, Data Segmentation and Inter-Rater Reliability

We first examined the raw footage and performed a micro-genetic, qualitative analysis of the video clips to segment the book-based interactive exchanges. To do this, we codified the behaviors of the children within second-by-second data frames using (ELAN, 2018). We defined nine codes (Table 5) related to communicative signs that were observed in the children (Moreno-Núñez et al., 2015, 2017, 2019). The nine codes were categorized in three groups: (1) uses of the book; (2) gesture production (to the educator, to the books or related to some meaning that was evoked by the books and/or their content); and (3) linguistic productions that accompanied both children’s uses and gestures (e.g., vocalizations, word utterances, and/or any combination or repetition of them). For this study, we excluded gestures that were directed to the researcher, who was therefore a non-participant observer.

**TABLE 5:** Analytical codes for children’s communicative signs.

**Uses of Objects**
**Unconventional Uses**
Unconventional uses do not relate to the social or cultural function of books. The child (C) uses the book in ways that denote that he/she is still unaware of its meaning or the social conventions around its use, e.g., sucking it, shaking it, licking it, and/or throwing it out. Children typically perform these uses whatever the object.
**Precursors to the Conventional Use of the Book**
C uses the book in a more directive and precise manner than in unconventional uses. However, it is not close enough to the conventional use, i.e., it foreshadows the emergence of the conventional use (based on Rodríguez and Moro, 1999). That is, C tries to open the book but fails, the book drops off her hands, she quits the task; C tries to turn the page unsuccessfully, opens or turns the pages with the book upside down.
**Conventional Uses**
C uses the book according to its social function, performing any of the following uses: C opens the book, turns the pages, or closes the book. These uses may be performed independently or while the teacher holds the book.

**Gestures ^1^**

**Ostensive**
The gesture is produced with an object that occupies the hand. That is, she gives or shows a book to the teacher (Moreno-Núñez et al., 2019).
**Indexical**
Sign and referent could coincide or not. The referent is present but distant, or the pointing gesture touches what is pointed to. This category includes pointing, touch-pointing, and reaching gestures. In other words, C touch-points to a picture in the book (Moreno-Núñez et al., 2019).
**Symbolic**
Gestures that represent people, objects, or events through hand or body movements or by facial expression. This category includes conventional gestures such as waving to say hello/goodbye, clapping, “where is it?” (raising the shoulders with the hand palms up), etc. (Murillo and Belinchón, 2013).
^1^ We coded children’s gestures during the use of the book and when the teacher shows C the book and/or reads to them.

**Linguistic Production ^2^**

**Vocalizations**
Includes both vowel and consonant vocalizations, simple and concatenated with prosody, i.e., babbling with consonant-vowel structure or vowel-consonant-vowel.
**Words**
Includes words and onomatopoeic sounds.
**Combinations**
Comprises the two previous categories while using the book, as well as the repetition of two of them.
^2^ We only considered those linguistic productions that accompanied the children’s uses of the book and/or their gestures.

All the video clips were initially coded by the first author. In addition, a second researcher with experience in early cognitive and communicative development assessed the suitability of the analytical categories for the observations under consideration. Both of them independently analyzed 18% of randomly selected video clips to calculate inter-rater reliability for children’s uses of the book, gestures, and linguistic productions. Inter-coder agreement was reached at 90.1%, which denotes an excellent level of reliability (Cicchetti, 1994).

#### Group Comparisons

We conducted a group comparison analysis to investigate whether there were statistically significant differences in the frequency of children’s production of communicative signs. We used IBM SPSS Statistics for Windows (v. 23) to calculate proportion comparisons and binomial tests. To this end, we considered as categorical variables the *Group* (control and experimental) and *Time* of data collection (pre-test and post-test) and as ordinal variables *Uses of the book*, *Gestures*, and *Linguistic production/combinations*. Because our study was aimed at analyzing the potential differences between the CG and EG, we ran a Kruskal-Wallis H test to compare our ordinal variables between the two instances of data collection (pre- and post-test).

We also compared the TADI scores by an analysis of variance with covariates (ANCOVA). The intergroup variable was *Group* (control and experimental), and the intragroup variable was *Time* of data collection (pre-test and post-test). *Age* and *Frequency of reading at home* were used as covariates. Results are presented and interpreted in the following section supported by data charts.

## Results

Second-by-second micro-genetic analysis of the interactions allowed for the identification of the variations in children’s uses of the book over the course of study, as well as of the diversity and complexity of their gestures and linguistic productions. In this section, we first present the group comparison analysis and then the results for the three main stages of the study: the pre-test, the 6-week book-reading intervention, and the post-test. For each stage, we focused on the children’s production of communicative signs—i.e., uses of the book, gestures, and linguistic productions—and highlighted some aspects of the specific mediation that the teacher made during the triadic interactions.

### Effect of the Teacher’s Mediation in Triadic Shared Reading Interactions

A goal of this study was to examine whether there was an effect of the teacher’s mediation in the triadic shared reading interactions with the children on the amount and diversity of the communicative signs that children used. To this end, we identified a total of 624 instances of communicative signs (uses of the book, gestures, and linguistic productions) for the quantitative analysis conducted.

#### Group Comparisons

**FIGURE 2:**
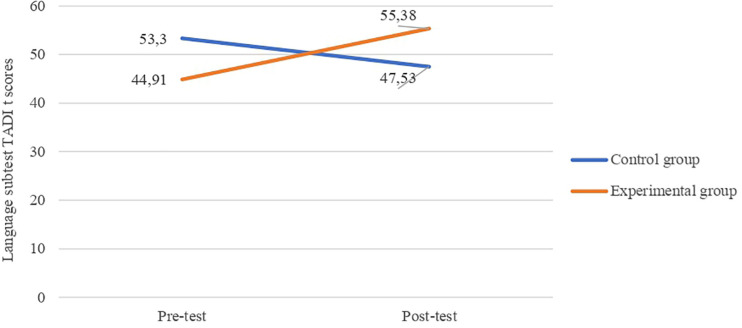
Average TADI scores according to the Group (control and experimental) and the Time of data collection (pre-test and post-test).

To investigate whether there were differences between the CG and EG in the two times of data collection, we conducted an analysis of variance for related samples while controlling for two variables: *frequency of reading practices* and *age*. The analysis showed that there was a significant effect of the intervention on children’s performance (*F*(1, 7) = 8.334; *p* = 0.023) (Figure 2). This means that differences in the language dimension of the TADI scores for the two groups of participants increased after the intervention, as compared with the pre-test scores.

**FIGURE 3:**
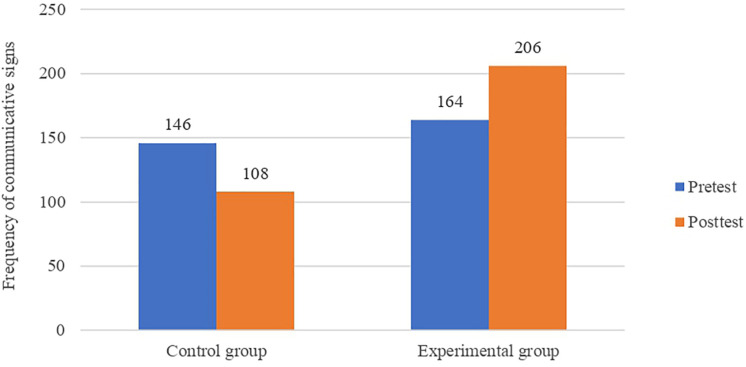
Frequency chart showing the total amount of communicative signs according to the Group (control and experimental) and the Time of data collection (pre-test and post-test).

During the pre-test stage, children in both groups produced a similar number of communicative signs, with no statistical differences according to the binomial test for equal proportions that we conducted (*p* > 0.05) (Figure 3). However, after the intervention, children in the EG almost doubled the quantity of communicative signs as compared to their control counterparts (*p* < 0.05).

Moreover, participants in the CG show statistically significant differences between the pre- and post-test (*p* < 0.05), contradicting our initial hypothesis, according to which we did not expect to find any difference. It is worth mentioning that the number of communicative signs performed by children in the CG decreased in the post-test stage.

**FIGURE 4:**
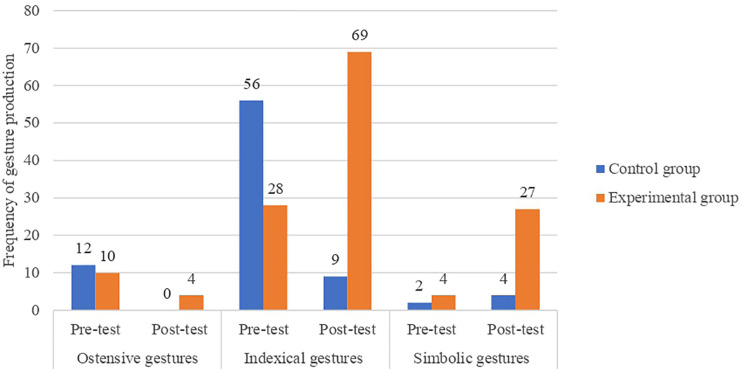
Frequency chart showing the different types of gestures accoridng to the Group (control and experimental) and the Time of data collection (pre-test and post-test).

We also conducted a proportion comparison analysis of the different types of gestures that children performed according to the group (control and experimental) and the time of data collection (pre-test and post-test). Both groups present similar frequencies of gestures at the pre-test stage (*p* > 0.05) but given the low frequency of observations in various combinations of these categories, significance data should be considered with caution. During the post-test, only children in the EG performed ostensive gestures, the frequency of which was statistically different from the CG (*χ*^2^(1, 26) = 4.052; *p* < 0.05). While, during the pre-test, children in the CG performed more indexical gestures than those of children in the EG, results showed that, during the post-test, children in the EG out-performed their control counterparts (*χ*^2^(1, 162) = 51.162; *p* < 0.05) (Figure 4). This could be due to the particularities of children’s developmental trajectories at this age.

After conducting the analysis of proportion comparisons of those gestures that were performed with any kind of linguistic production, we found that the EG presented a larger proportion of indexical gestures with vocalizations (*χ*^2^(1, 30) = 7.033; *p* < 0.05) during the post-test stage (60.0%) than children in the CG (13.03%). All types of symbolic gestures accompanied by vocalizations were observed only during the post-test session of the EG. However, the low frequency in the diverse combinations of these categories does not allow for establishing any statistical significance.

**FIGURE 5:**
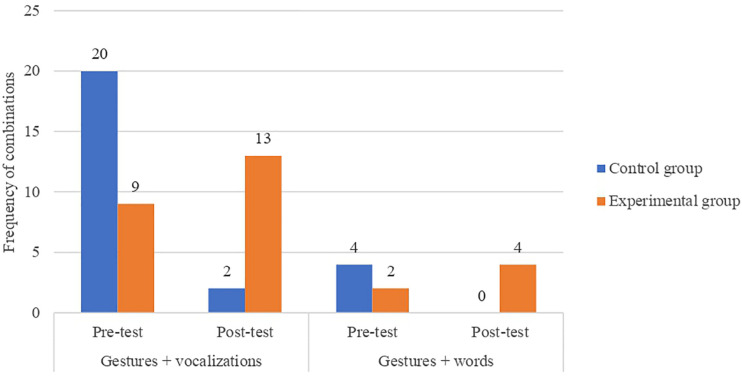
Frequency chart showing combinations of gestures and linguistic productions according to the Group (control and experimental) and the Time of data collection (pre-test and post-test).

The EG also presented a larger general proportion of gestures + vocalizations during the post-test than the control group (*χ*^2^(1, 44) = 12.239; *p* < 0.5) (Figure 5). This is particularly relevant since the four types of gestures observed during the post-test, when accompanied by onomatopoeias or words, were performed by children in the EG (*χ*^2^(1, 10) = 4.444; *p* < 0.05). However, given the low frequency of observation for these categories, our results suggest some degree of caution.

### Pre-test Stage

During the pre-test stage children in the CG performed a total of 84 uses of the books, while children in the EG performed 129. We analyzed these instances according to the type of use.

First, we found that four of the five children in the CG performed a total of 16 *unconventional* uses. Out of these, 12 were performed by a 7-month-old boy. Conversely, we observed 59 unconventional uses by participants of the EG, performed by four of the six children in this group. It is striking that the EG child paired with the higher performer in the CG—who was 8 months old—performed a total of 45 unconventional uses.

Regarding the *precursors* to conventional uses, children in the CG performed 10, while in the EG the frequency was 5. In the CG, only three of the participants were observed performing precursors. The most frequent form was an unsuccessful attempt to open the book in a conventional position (6 in total). As for the EG, only two out of the six children used the book in this nearly socially conventional manner. The most frequent example, however, was only observed twice, when children tried to open the book upside down.

When we analyze the *conventional uses* of the book, we find that children in the CG performed a total of 58 conventional uses with all the three books that were available at this phase, a frequency that was close to that observed in children from the EG (65 uses). These uses were observed in four children of each group, control and experimental. Participants of both groups performed all three types of conventional use that we identified: they opened the book, turned the pages to contemplate its pictures, and closed it.

While younger children in both groups (4 and 5 months of age) were not observed performing any conventional uses, children over 12 months frequently did so. The 7- and 8-month-olds (from the CG and EG respectively) performed a single conventional use during the pre-test phase. The 9- month-old child in the EG did not perform any conventional use at this time.

We also analyzed children’s gesture production, finding that four out of the five children in the CG performed a total of 70 gestures during the pre-test. Most of these were of an indexical nature and were observed in a 13-month-old boy (19 indexical gestures) and a 14-month-old girl (37 indexical gestures). These children were the only ones who performed symbolic gestures as well. Ostensive gestures, giving or showing the book to the teacher, were used by children from 7- to 14-months-old for communicating something about the book. The 5-month-old boy was the only child who did not perform any type of gesture.

In the EG, five of the six participating children, ranging from 8 to 15 months of age, performed a total of 42 gestures. Indexical gestures, including touch-pointing gestures, were observed in four of these participants, although the majority of them were performed by a 15-month-old girl. Similarly to what we observed in the CG, symbolic gestures in the EG were only performed by a 12-month-old girl and a 14-month-old boy, who were the pair of the same age mentioned above in the CG results.

Out of those four from the CG that performed uses of the book and gestures, *linguistic productions* were observed in three children. These three accompanied some of their uses of the book with linguistic productions, especially at 13 and 14 months of age. Out of the total of 40 linguistic productions identified, the most frequent type was children’s vocalizations, which were observed together with other communicative signs in a total of 29 instances, followed by words (including onomatopoeias), with 9 utterances observed. Lastly, we only observed one use of two different onomatopoeias together, which we defined as a combination.

Among the participants in the EG we found that five children accompanied their uses and gesture production with some type of language in 26 occasions. Vocalizations were performed by children from 8 to 15 months of age, and were more frequent than those observed in children in the CG. Unlike the CG participants, where three children were observed combining communicative and linguistic signs, there were only two in the EG. None of the participants in the EG performed sign combinations of two or more vocalizations and/or words.

Micro-genetic analysis showed that children do not only accompany their uses and/or gesture production by some type of linguistic production, but also that they combined uses and gestures, adding sometimes a vocalization, a word or a combination of both. We observed six combinations of use and gesture in the CG, performed by the 14-month-old girl. They were mainly autonomous conventional uses (open the book, turn the pages, or close the book), i.e., performed with no support from the teacher. All these conventional uses were accompanied by touch-pointing gestures to indicate a character or animal in the book several times, i.e., with communicative redundancy. The girl did not look at her teacher at any time, which could indicate that these were self-directed gestures. She also performed a use combined with a touch-pointing gesture and a vocalization, adding a linguistic production to what she had been doing before.

Within the EG, only two girls (14 and 15 months of age) performed a total of five combinations of conventional use and gesture. In all cases, the girls performed the uses autonomously: they held the book, opened it (though sometimes it was upside down), turned a page, and closed them. Four of those gestures that accompanied these uses were touch-pointing gestures to indicate something that was represented in the book. In addition, we identified a gesture that was considered symbolic, as shown in Observation 1.

**Observation 1**
Girl 2. Experimental Group. Age: 1; 2 (9)
The child is looking at the pictures that the teacher has pointed out in the book and labeled as “the dad and the son.” The teacher says to the girl, “Look, how pretty!” as she *touch-pointed the picture of both characters*. The child hits the picture several times, performing an *unconventional use* of the book. When she stops, she looks at her teacher and *closes* the book. Her teacher then says “a little kiss? Here, give me a little kiss”. The child, who is contemplating the book’s cover, *brings it closer to her face and kisses the character* that is represented on it.

Just one of the boys in the EG, of 12 months of age, performed two uses combined with pointing gestures and an onomatopoeia and a vocalization, respectively (Observation 2).

**Observation 2**
Boy 5. Experimental Group. Age: 1; 0 (13)
The child *turns the book pages* and *stops to contemplate* the picture of a “mommy dog and her puppies.” The teacher, noticing the dogs in the picture, says “woof.” The child *answers with a vocalization and looks at his teacher smiling.* She smiles back and asks, “What does it say?” as she points at one of the dogs. The child answers *“woof,”* and the teacher congratulates him and says, smiling, “That’s it, very good.” The child then *touch-points to the pictures* of the dogs in the book.

In order to discard any statistically significant differences at the starting point of the study, we compared TADI scores from both the CG and the EG. We conducted a Mann-Whitney test, which indicated that there were no significant differences between the groups (*U* = 11.00; *p* = 0.459). These results support using pre-test scores for comparisons with no need to control for this variable.

### Intervention Stage

**FIGURE 6:**
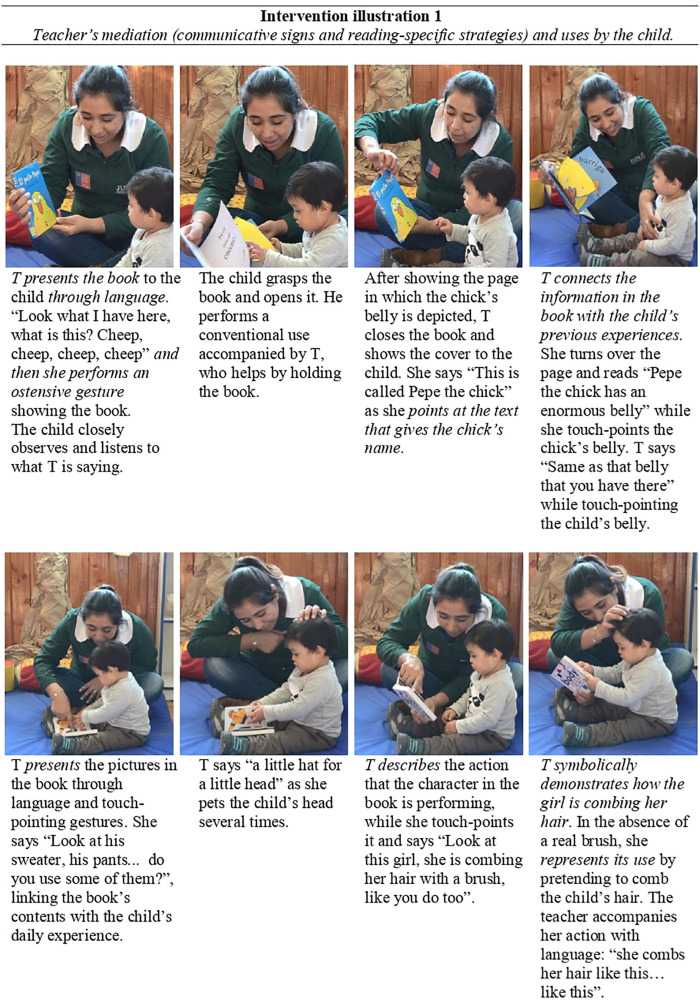
Intervention illustration 1-Boy 5 and his teacher (T) reading “El pollo Pepe” [Pepe the chick] (above), and “Body” (below).

In order to exemplify the richness and variety of the communicative signs that the teacher used during her initiatives of triadic interactions with children and books, we have selected some representative instances of both one-to-one and group-reading interactions. Figure 6 illustrates two examples of one-on-one shared reading interactions with two different books, as well as the teacher’s mediation through her use of communicative signs and specific strategies for reading promotion.

**FIGURE 7:**
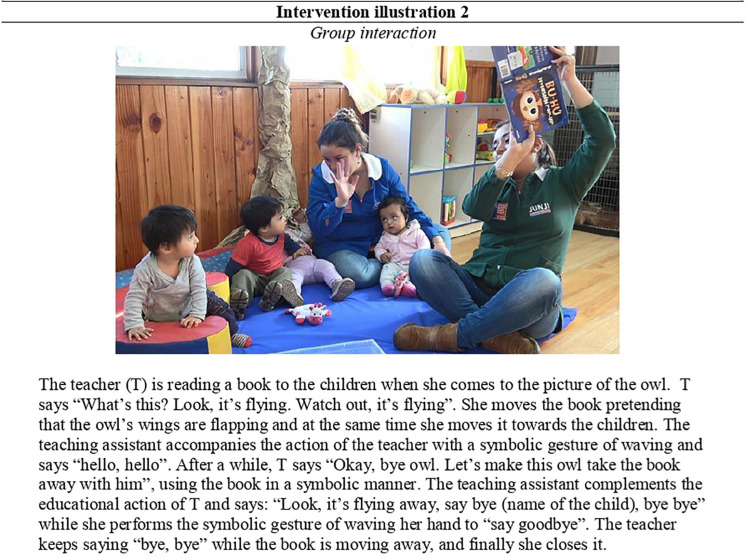
Intervention illustration 2-Teacher’s symbolic uses of the book “Buho” [Owl], which is a pop-up book.

An example of group interactions is shown in Figure 7 for illustrative purposes.

### Post-test Stage

During the post-test phase children in the CG performed a total of 99 *uses of the book*, 15 more than in the pre-test. Children in the EG performed 118 uses, reducing in 11 cases from those observed during the pre-test. The variety of uses of the book performed by children in the EG were of diverse semiotic complexity, ranging from unconventional to conventional uses. Unconventional uses reduced in both groups during the post-test phase. While in the CG we observed a single child (17 months of age) performing a total of five unconventional uses, the eight instances identified in the EG were performed by three different children. The child who performed 45 unconventional gestures during the pre-test performed only 3 in the post-test. In the EG, the youngest child (6 months of age), who did not perform any uses at the pre-test, performed a single *unconventional* use of the book at the post-test (she put the book in her mouth to bite it). She did not perform any other use during the post-test.

Following the complexity rationale, we also observed slightly more complex uses, which we refer to here as *precursors* (i.e., of the conventional use). These uses still require some adjustments in order to be effectively performed according to the conventional use. For example, children sometimes tried to open the book from its spine, or they opened the book and turn the pages, but while holding the book upside down. These precursors were performed by two out of the five children in the CG, with a total of seven precursors to the conventional use of the book. It is striking that these two children were also the oldest in this group, both being 17 months of age. The two girls of this age in the EG performed the same number of precursors. However, in the EG we also observed precursors in children from 11 to 15 months of age. After the child’s precursor use, the teacher either continued with her dialogue or with the questions that she was already posing, as part of the conversation that guided the interactions. However, there were also occasions in which the teacher noted, for example, that the book was not in the correct position, such as in the example depicted in Observation 3.

**Observation 3**
Girl 1. Experimental Group. Age: 1; 5 (0)
The child enters the room and *immediately sits in the corner where they usually conduct the reading sessions.* The researcher piles the three books on the mat, and the cover picture of the book that is on top stays upside down from the child’s perspective. The child makes some unintelligible but intonated jargon. When the researcher left the books on the floor, the child stood up while her teacher says, “There are the books,” at which the girl laughs. She then takes the books while her teacher gets closer and says, “Come, come, there you go, take them, OK,” helping the child to grasp them. The girl drops the books, while the teacher says, “Let’s sit down here together” and indicates, *touch-pointing with her hand twice*, the place where she wants the child to sit down. They both sit, and the teacher places the books again, on the mat (close to the child) in the same position that they were originally. She says, “OK, which one are we going to read?” The child *takes one of the books, turns it over, and looks at the back cover*. The teacher says, “That one? The one with the animals?” The child turns to her teacher and responds with some unintelligible, intoned jargon. The teacher answers, “Yes? OK.” The child *opens the book upside down and looks at its pictures.* The teacher says, “Here, who…?” but then says, “It is upside down,” while accompanying her language with palms up and her face in a questioning attitude. The child *carefully looks at the pictures and smiles*. The teacher says, “Let’s see,” and tilts her head to look, while the child *looks at her smiling*. The teacher takes the book and turns it the right way around, saying, “Like this, look. This is the way.” She places the book in the child’s hands and says, “What is this, is it a puppy?” as she points at the dog’s picture. The child looks at the researcher and says something unintelligible.

**FIGURE 8:**
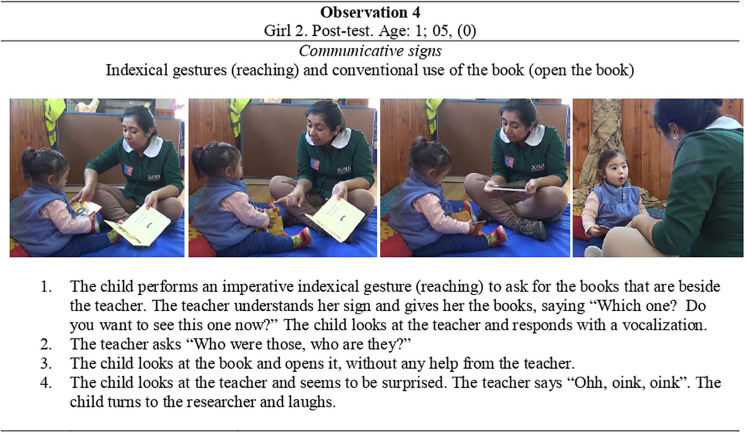
Observation 4-Girl 2 and her teacher at the post-test interaction (a).

Lastly, we identified several *conventional uses* of the book performed by children in both groups. Every participant except the 6-month-old babies performed conventional uses during the post-test. Children in the CG performed 87 conventional uses in total, while the EG frequency was 91. As with those which we observed during the pre-test, all three types of conventional uses were observed, i.e., open the book, turn one or more pages, or close the book (as in concluding its use). Children tended to be more active in the interaction when the teacher was reading to them: they could engage in their own uses while the teacher helped them by holding the book from below. For instance, in these circumstances they were able to turn the page or contemplate the book pictures in a shared reading with the teacher. In other words, the teacher provided a physical support, guiding the reading interaction and facilitating the children in getting used to using the book by themselves. At other times, however, the children autonomously used the book, taking it on their own initiative, opening it, and looking at the pictures, as shown in **Observation 4** in Figure 8.

Children also performed several *communicative gestures* while using the books during the interactions. According to their semiotic complexity and increasing distance between sign and referent, the simplest gestures that we observed were *ostensive* gestures, those in which the sign and the referent coincide (i.e., the referent occupies the hand). For instance, children often performed ostensive gestures to show or to give the book to the teacher, either in an imperative manner (requesting for some action from her) or just to share attention to the book or its pictures. Children also interacted with the teacher about the book or the shared reading situation by using *indexical* gestures. Among these, the most frequent gestures observed were touch-pointing gestures, followed by distal pointing and reaching gestures.

**FIGURE 9:**
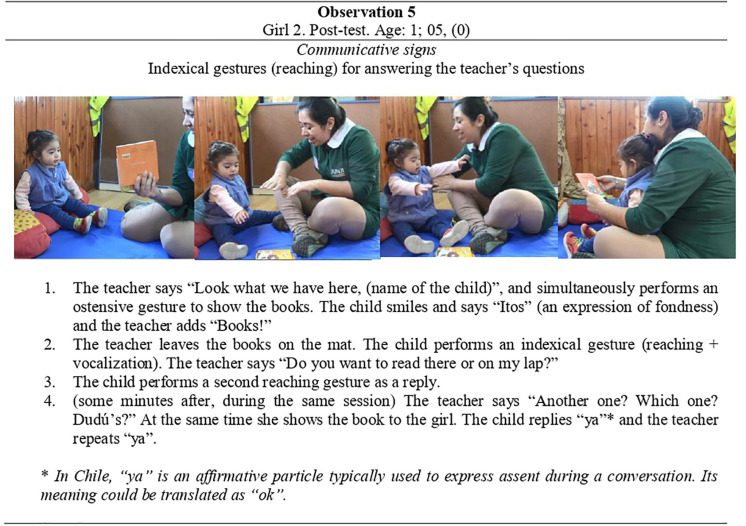
Observation 5-Girl 2 and her teacher at the post-test interaction (b).

Participants in the CG decreased their gesture production at the post-test, totaling 13 instances. These productions were indexical and symbolic, indexical gestures being the most frequent. Gesture production in the CG was only observed in three out of the five children, from 16 to 17 months of age. In the EG, not only gesture production but also the number of children that performed them increased. A total of 100 gestures were observed in five out of the six children in this group (from 11 to 17 months of age). Among them, indexical gestures were again the most frequently performed, followed by symbolic gestures, which increased to 27 instances. Ostensive gestures were the least observed, and only in some of the children. **Observation 5** in Figure 9 illustrates how the child not only used indexical gestures to communicate with her teacher, but also combined them with words to highlight what she wanted to read and how she wanted to do it.

**FIGURE 10:**
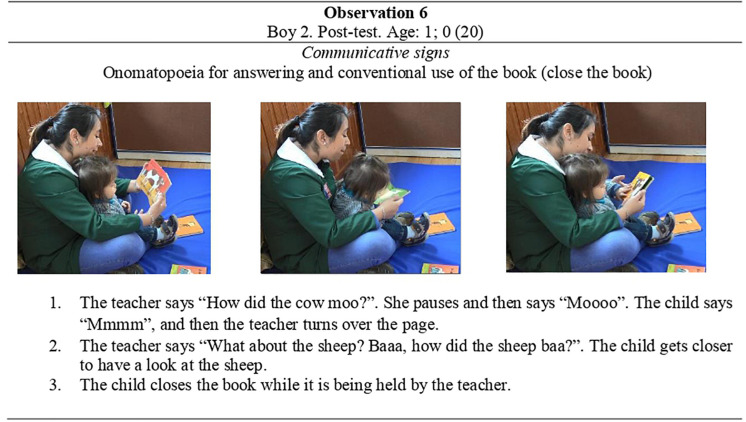
Observation 6-Boy 2 and his teacher at the post-test interaction.

The *linguistic productions* that were observed in this study were widely diverse and age-dependent. First, the same three children in the CG who made some linguistic production during the pre-test were also the ones that used them at the post-test stage. However, their frequency was reduced to 17 instances. Conversely, linguistic productions by children in the EG increased to 37 utterances. Among these productions, in the CG the most frequent type of linguistic production were words (9 instances), followed by vocalizations (7 observations). In the EG, the most frequent linguistic type was vocalizations (23 instances), followed by words (8 instances) and combinations of different linguistic productions (6 examples). Combinations were rather rare in the CG. **Observation 6** in Figure 10 illustrates how children incorporate these signs to the interaction.

Children in this study also performed combinations of diverse communicative signs during the triadic interactions. In the CG, the girl that performed six combinations of use and gesture during the pre-test only performed it once during the post-test, as was also the case with a 16-month-old boy. In both cases, the children were turning the pages of a book that was held by the teacher. Accompanying gestures were a touch-point and a symbolic gesture to represent little birds. Only one of the children in this group performed a triple combination of use, gesture, and linguistic production (at 17 months of age). Regarding the EG, the girl who initially performed four combinations of use and gesture at pre-test, did not perform any during the post-test. There were, however, eight combinations of use and gesture, six performed by a 15-month-old boy, and two by a girl of 17 months of age. Complex combinations of uses, gestures, and linguistic productions were observed in three children in this group, from 15 to 17 months of age.

## Discussion

In this experimental study our aim was to determine whether there is an effect of teachers’ mediation (communicative signs and reading strategies) on children’s production of communicative signs during triadic shared reading interactions (teacher-child-book). In addition, we characterized the communicative signs that were observed in children during their first year and a half of life in book sharing interactions with their teacher.

Proportion comparisons resulted in evidence that participating in one-on-one and group interactions during a six-week intervention had a positive effect on the number of communicative signs that children in the EG performed when communicating with their teacher. Although a period of six weeks might be too short for observing changes in development, our findings coincide with those of Valdez-Menchaca and Whitehurst (1992). These authors also found differences that favor the EG after participating in a similar six-week intervention. However, these differences were particularly related to language assessment of children over 2 years of age. Our findings suggest that children become able to communicate with their teacher very early through shared reading interactions with books, even when they are still in a developmental stage at which language is only just beginning to emerge. Communication occurs based on several sign systems such as gestures and uses of the book, as well as smiles and gazes that strengthen the connection between the child and adult.

Nonetheless, not all the children increased their production of communicative gestures in a similar proportion at post-test. This was an expected outcome because of the varying ages of our participants and the consideration that the teacher did not use a standard script that was exactly the same for every child. Instead, she adapted her mediation to the developmental characteristics of each child, and to their actions with the books. While four of the children in the EG increased the amount of communicative signs they produced, the other two decreased them. In fact, one of these two considerably reduced his unconventional uses, which is a trend that was observed in both the CG and EG at the post-test. Given that during the first year of life children perform several types of uses of objects (Rodríguez and Moro, 1999; Moreno-Núñez et al., 2017, 2019), perhaps the physical properties of the pre- and post-test set of books (size, shape, weight) might influence children to use them in a different proportion, for example, to perform fewer uses with some of them because of additional exploratory difficulties. An additional potential explanation could lie in the research setting, as children often sat on the teacher’s lap, which could have reduced their possibilities for acting directly with the books. In any case, offering a diverse repertoire of books for the different sessions enabled the teacher to better adjust to the specific needs of the children and to their developmental characteristics.

The book acted as a referent of communication across all the phases of this study, which allowed children to gain significant knowledge about the object itself, but also about several referents that were represented in the books (e.g., animal pictures, stories whose characters experienced similar routines to those of the children in their daily lives, etc.). In addition to their uses of books, children also performed varied gestures of different levels of semiotic complexity across the study. That is, books can be shown or offered, looked at and listened to. Children can point at the pictures, or they might represent something that the books evoked in them. Children reached progressively more complex agreements with adults through shared interactions, in which the book acted as a bridge, or a link between them and their teacher. Books turned into a core element for cognitive and communicative development in young children and the main focus of the communicative interchange, but we cannot ignore the fact that books are cultural objects well, and as such they involve several and diverse representations that catalyze their communicative possibilities (Contin and Rodríguez, in press; Rossmanith et al., 2014).

The diversity of communicative signs supports the idea that the use of a book is not evident for young infants and toddlers, as are other objects that are loaded with cultural meaning (Rodríguez et al., 2018). Object use requires a systematic and semiotically mediated action by an adult so that children can progressively internalize the public meanings that a book involves. Children gradually understand the cultural complexity of the book through its use and become more efficient with practice, which is often mediated by the teacher. For this reason, unconventional uses sometimes co-occurred with more complex uses that involved a higher degree of convention. These first approaches to the use of a book (unconventional uses and precursors) may constitute the basis of conventional uses, which emerge thanks to the extensive network of multimodal signs that are activated during shared book reading.

Communicative signs, however, are not restricted to gestures and uses of objects. In fact, as occurred with the combinations of gestures and uses just discussed, book-sharing situations invite the participants (adult and child) to incorporate a wide variety of linguistic productions. The simplest vocalizations observed in the children did not abruptly disappear, rather they coexisted with more complex linguistic forms, such as words (Murillo and Belinchón, 2013). The results also pointed to individual differences among children. Even at the same age, children’s communicative signs differed in terms of amount and complexity. This could be due to individual rates of development, i.e., the variability that underlies the emergence of language in children, which could happen anytime from 10 to 18 months (Mariscal and Gallo, 2014), but it could also be the result of interaction. For instance, our findings show that children in the EG performed more uses or gestures combined with any type of linguistic production than children in the CG. Moreover, the frequency of use and gesture combinations of the EG was equal to or higher than those performed by children in the CG.

The communicative possibilities that a book offers are multiple because of the numerous additional referents that are represented in its pictures. This fact could have some relevance in those cases of children that overfocus on certain types of uses and gestures, which could explain some of the differences that were observed between the two groups. For instance, during the pre-test, children in the CG performed more indexical gestures than their EG counterparts. This could be because children use gesture performance to help themselves contemplate whatever is represented in the books (Bates et al., 1976), which could explain the post-test decrease due to children’s previous experiences with the book in question. Children soon realize that they can use several communicative signs for communicative purposes, not only with themselves, but also with their teacher.

Focusing on the variety of children’s communicative gestures, we find it striking that only children in the EG performed ostensive gestures to give or to show during the post-test observations. However, they frequently performed indexical gestures, which have been a major focus of developmental research, where distal pointing gestures are *the* gestures for communicating about shared referents (Liszkowski et al., 2007; Tomasello et al., 2007). Children in the EG frequently used the book together with different combinations of vocalizations, words, or repetitions of them, which could be due to the reading experience that they acquired through the intervention. They were exposed to a wide variety of signs and new words during book sharing interactions with their teacher, both in the one-on-one and the group interventions.

While we must be cautious when comparing proportions between groups because of the low frequency of certain combinations of communicative signs, findings are encouraging and support the continuing promotion of book sharing interchanges at ECE centers from the first year of life. Ideally, teachers should foster one-on-one interactions in which they dedicate individual time to interpreting the communicative signs of the child. That would allow them to use their observations to plan particular actions based on the use of books that could foster new and more complex ways of communication with the network of meanings that children have developed during the interactions.

The set of hypotheses that were supported (one, two, and four) allow for claiming that the use of books has a favorable effect on children’s production of communicative signs between 4 and 15 months of age during shared book reading interactions with their teacher. Findings showed that the group of children that participated in the six-week intervention (EG) performed more communicative signs than those children in the CG. These results validate our second hypothesis and add evidence for the positive effect of using books as a means to promote young children’s communication. Additionally, the frequency of communicative signs performed by children in the CG decreased during the post-test, refuting our second hypothesis.

Regarding the decrease in the communicative signs of children in the CG from pre- to post-test, results could have been influenced by the fact that one of the children in this group spent less time interacting with the teacher during the post-test filming. The child did not attend school during the whole six-week period of the intervention and had just came back for the post-test data collection. As a result, he could have been still readapting to the school routines and thus got tired of the activity earlier than the rest of the children. Nonetheless, we consider that this decrease of communicative signs was primarily affected by the absence or the lack of participation of children in the CG in one-on-one and group reading sessions under the intervention design. Both one-on-one and group communicative interactions with the teacher were used to intentionally show, describe, and link the content of different books to the children’s previous experience (either at home or at school). The teacher used a whole set of varied signs that are understandable to the children. In addition, her mediation was not solely based on communicative signs and language (which helped to organize the interaction), but also on specific reading-oriented strategies. During the intervention, children in the EG had access to books that were not available for pre- and post-test assessment, which could also have increased their possibilities for communicative interchanges with the teacher.

All in all, the mediation of the teacher could have helped to increase the amount and variety of communicative signs that children performed during the post-test. This result is consistent with the observed changes in the TADI scores; the TADI has been shown to be a very effective and contextual tool to complement the assessment of language development in Chilean children.

Among the limitations of this study we should mention that the limited sample size—a fact beyond doubt—reduced the power of the statistical tests that were used. For the same reason, it is possible that there would be statistically significant differences that could not be detected. Hence, it would be necessary to expand the sample size in future studies in order to overcome this limitation. Another limitation of this study is that our research design does not allow for depicting the effect of every factor involved in the intervention, i.e., the triadic interaction through semiotic mediation, and the specific strategies that the teacher used for mediating shared reading and communication. An additional difficulty has been the little control that we had over children’s attendance at school in all stages of the study. Being very young infants, they were often absent because of health-related issues. However, a prolonged absence could interfere in the amount of interaction time that a particular child had with his or her teacher, as well as in their readjustment to the school routines and to the filming situation. Despite our efforts to control certain variables, such as the frequency of reading habits at home and the age of the participants, it is possible that we have not considered other factors (e.g., the access to and use of electronic devices) that could potentially influence the children’s production of communicative signs. Last, it is also a limitation that we did not control for the duration of the reading episodes, which could have influenced the amount of communicative gestures that children performed.

Considering the importance of materiality for the earliest stages of development, future research could compare the possible variations in children’s communicative signs according to the different types of books (e.g., interactive books and picture books). This could contribute to our understanding about whether or not the book type affects the promotion of better communication between children and teacher. In addition, research could include other analyses that specify, for instance, what kind of linguistic productions are typically combined with each type of signs, deepening the analyses and descriptions that we have reached in this study. It would also be interesting to explore the communicative functions that underlie children’s gesture production, for example, by conducting a comparative analysis that reveals intra-subject differences beyond the statistical tests that were used for this study.

Future studies should investigate whether or not the nature and/or the characteristics of objects influence children’s performance of ostensive gestures. It is possible that the set of books used for the pre- and post-test stages were not easy to grasp/manipulate for the children (because of their shape and size). This could explain the fact that almost no uses were observed in the youngest children, who were six months of age at the post-test. It could be relevant to also include alternative activities for children in the control group, so that we could minimize the effect of time and type (one-on-one and group activities) of teacher-child interaction. Finally, studies focused on analyzing how the teacher specifically mediates in triadic shared reading interactions will be of interest for a more detailed and thorough exploration of any progression or incremental development in the communicative signs that children produce after participating in the intervention.

## Conclusion

The conventional uses of a book are not evident, so a child’s comprehension requires the semiotic mediation that adults bring to interaction. This study describes children’s first approaches to books in an ECE center, a book being a complex cultural object in which different modes of signification occur (e.g., the object, the text, and the pictures). While certain uses such as opening a book, turning its pages and closing the book to conclude its use are conventional and efficient, they are just the starting point for more complex uses that children will keep learning and mastering over the years. These uses, as well as others that precede them, allow children to keep building new meanings around everyday objects, on which they often rely for other supports and signs.

This study confirms the evidence provided by previous studies on the impact of book sharing interventions on children’s communicative abilities. Prior research focused either on the study of children over three years old or with younger children in interaction with their parents. In this article, we have discussed some of the benefits that book sharing practices could have for the cognitive and communicative development of infants in triadic interactions with their teacher. The better abilities observed in children in the EG during the post-test, both in terms of the performance communicative signs and in the TADI scores, should encourage nursery teachers to foster shared reading practices with children from the first months of life, as soon as they entered the ECE system. Instances of triadic interaction promote the understanding of the uses of complex sociocultural objects, allowing for increasing the amount of communicative exchanges and promoting children’s construction of meanings and the acquisition of communicative, linguistic, and non-linguistic signs that are increasingly diverse and complex.

## Data Availability Statement

The datasets generated for this study are available on request to the corresponding author.

## Ethics Statement

The studies involving human participants were reviewed and approved by Scientific Ethics Committee for Social Sciences, Arts and Humanities of the Pontificia Universidad Católica de Chile (i.e., the Institutional Review Board). Written informed consent to participate in this study was provided by the participants’ legal guardian/next of kin. Written informed consent was obtained from the individual(s), and minor(s)’ legal guardian/next of kin, for the publication of any potentially identifiable images or data included in this manuscript.

## Author Contributions

KC is the Principal Investigator for this project. She was in charge of conceptualizing the study, as well as coordinating the contact with the participants and data collection. She did the initial coding, literature review, qualitative analysis and drafted the report of the results. AM-N contributed to refining the coding system and performed the second round of coding for inter-rater reliability purposes. She contributed to this article by conducting an exhaustive literature review, as well as writing down the initial drafts and revising different versions of the manuscript. EM-Z helped to design the research study and to organize the dataset for further analysis. He conducted the quantitative analyses that are included in this article and revised different versions of the manuscript. All authors contributed to the article and approved the submitted version.

## Conflict of Interest

The authors declare that the research was conducted in the absence of any commercial or financial relationships that could be construed as a potential conflict of interest.
